# Reducing co-occurring alcohol-related consequences and depressive symptoms among university students

**DOI:** 10.1186/1940-0640-7-S1-A45

**Published:** 2012-10-09

**Authors:** Tibor Palfai, Timothy Ralston, Leslie Wright, Timothy Brown

**Affiliations:** 1 Department of Psychology, Boston University, Boston, MA, USA; 2 Department of Clinical Psychology, Boston University, Boston, MA, USA; 3 Psychological Services Center, Boston University, Boston, MA, USA; 4 Center for Anxiety and Related Disorders, Boston University, Boston, MA, USA

## 

Hazardous drinking and depression represent two major sources of harm and dysfunction for university students. This study sought to determine how best to address these frequently co-occurring conditions for students who are not seeking treatment. Participants were recruited through internet ads and screened by phone and in-person to participate in a study on alcohol use, stress, and student life. Thirty university students (aged 18-22) with hazardous drinking who exhibited depressive symptoms were randomly assigned to one of two conditions: assessment only or goal systems intervention (GSI). The GSI is a 3-session motivational intervention that addresses risks and processes of change related to alcohol and depression within an integrated framework. Students completed assessments of depressive symptoms, alcohol consequences, and heavy drinking episodes at baseline, three months, and six months. Given the small sample size of this pilot trial, our main goal was to provide effect-size estimates. Results of the conditional latent growth analysis for alcohol-related negative consequences showed a significant medium-to-large effect of the intervention (f-squared = 0.27). Similarly, analysis showed that the intervention resulted in a medium to large effect on reduction of depressive symptoms (f-squared = 0.323). There was little evidence of intervention effects on change in heavy drinking, as students in both conditions exhibited a significant decline in heavy drinking episodes over time. These results suggest that GSI may be an efficacious intervention to reduce alcohol-related harm and depressive symptoms among university students in opportunistic settings.

